# Conspicuity of malignant pleural mesothelioma in contrast enhanced MDCT – arterial phase or late phase?

**DOI:** 10.1186/s12885-021-08842-0

**Published:** 2021-10-26

**Authors:** Lukas Luerken, Philipp Laurin Thurn, Florian Zeman, Christian Stroszczynski, Okka Wilkea Hamer

**Affiliations:** 1 Department of Radiology, University Medical Center Regensburg, Franz-Josef-Strauss-Allee 11, 93053 Regensburg, Germany; 2 Center for Clinical Studies, University Medical Center Regensburg, Regensburg, Germany

**Keywords:** Malignant pleural mesothelioma, Contrast enhanced MDCT, Chest imaging, Oncologic imaging, Retrospective study

## Abstract

**Background:**

To determine if late phase is superior to arterial phase intraindividually regarding conspicuity of MPM in contrast enhanced chest MDCT.

**Methods:**

28 patients with MPM were included in this retrospective study. For all patients, chest CT in standard arterial phase (scan delay ca. 35 s) and abdominal CT in portal venous phase (scan delay ca. 70 s) was performed. First, subjective analysis of tumor conspicuity was done independently by two radiologists. Second, objective analysis was done by measuring Hounsfield units (HU) in tumor lesions and in the surrounding tissue in identical locations in both phases. Differences of absolute HUs in tumor lesions between phases and differences of contrast (HU in lesion – HU in surrounding tissue) between phases were determined. HU measurements were compared using paired t-test for related samples. Potential confounding effects by different technical and epidemiological parameters between phases were evaluated performing a multiple regression analysis.

**Results:**

Subjective analysis: In all 28 patients and for both readers conspicuity of MPM was better on late phase compared to arterial phase. Objective analysis: MPM showed a significantly higher absolute HU in late phase (75.4 vs 56.7 HU, *p* < 0.001). Contrast to surrounding tissue was also significantly higher in late phase (difference of contrast between phases 18.5 HU, SD 10.6 HU, *p* < 0.001). Multiple regression analysis revealed contrast phase and tube voltage to be the only significant independent predictors for tumor contrast.

**Conclusions:**

In contrast enhanced chest-MDCT for MPM late phase scanning seems to provide better conspicuity and higher contrast to surrounding tissue compared to standard arterial phase scans.

## Background

Asbestos, once widely used in construction and engineering due to its favourable chemical and physical properties, is known to cause a variety of diseases, one of them being malignant pleural mesothelioma (MPM). Even after banning asbestos, long latency periods of several decades between exposure and onset of disease as well as ongoing unavoidable exposure, for example when renovating old buildings, are the reasons why asbestos-related diseases like MPM are and will still be of clinical relevance for many more years. Disease-peak for many European countries is predicted to be between 2015 and 2030 with about 1600 predicted deaths during the peak year in Germany alone [1, 2].

Prognosis for patients with MPM is generally poor, but studies have shown that survival time correlates with tumor stage at time of diagnosis [3, 4]. Hence, early detection of MPM is of utmost importance for patient prognosis. Contrast-enhanced multi-detector-computed-tomography (MDCT) of the chest is the imaging modality of choice for detection and staging of MPM [5–10]. Alternative imaging techniques are magnetic resonance imaging (MRI) and positron emission tomography (PET)-CT. These modalities are not part of routine diagnostic work-up for every patient with suspicion of MPM, mainly due to lack of availability of MRI and in case of PET-CT due to radiation dose reduction issues but play an important role in staging and follow up of complex cases where contrast-enhanced MDCT alone is insufficient [5–10].

Empirical data and our own experience suggest that a longer scan delay after intravenous administration of contrast agent compared to the usual around 35 s delay used in most routine protocols for chest imaging might be superior in terms of detecting pleural pathologies in chest MDCT [7]. Study data proving this thesis, though, is scarce [11–13]. Recommendations regarding scan delay are inconsistent between guidelines with for example the British Thoracic Society preferring 60 s of delay and the German Radiology Society recommending arterial phase scanning [14, 15]. There is only one study that compares tumor visibility on MDCT intraindividually using different scan delays, but that study grouped several pleural malignancies together with MPM representing a minority of cases. Moreover, the authors focussed on subjective image evaluation and density measurements were evaluated for tumor lesions only and not for the surrounding soft tissue [16], but tumor conspicuity not only depends on the absolute density of the tumorous lesion but also on its contrast, compared to the surrounding tissue.

The aim of the current study was to compare the conspicuity of MPM on contrast enhanced chest MDCT in arterial phase and late phase intraindividually subjectively and objectively to determine, if late phase is superior to arterial phase.

## Methods

### Patient selection

This retrospective study was approved by the institutional review board and the independent ethics committee at our institution. Patients were identified by means of a full-text database query of all MDCT-scans conducted in our tertiary care university medical center between January 1, 2003 and April 31, 2015, using the term “*mesoth*” in the Radiological Information System (RIS, Nexus.medRIS, Version 8.42, Nexus, Villingen-Schwenningen, Germany). Additionally, patients who were registered in the local interdisciplinary tumor-registry with the diagnosis of MPM were added to the results. All in all, 760 patients fulfilling these criteria were identified.

These 760 patients were further analysed by screening patients’ charts and full radiology reports and by reviewing CT images in our Picture Archiving and Communication System (PACS, Syngo Imaging, Siemens, Erlangen, Germany) according to the following criteria:

#### Exclusion criteria

- False positive database-hits like patients with peritoneal mesothelioma
- False positive database-hits like “results not in keeping with pleural mesothelioma”

#### Inclusion criteria

- A contrast enhanced chest CT in standard arterial phase and an abdominal CT in portal venous phase (late phase, scan delay ca. 70 s) with overlapping fields of view were available.
- The maximum time between the two scans was less than 50 days in case MDCTs were not done on the same day
- No chemotherapy or thoracic surgery was done between the MDCT scans in case chest and abdominal MDCTs were not done on the same day
- The primary tumor was at least partially located in the overlapping field of view of arterial and late phase images
- Tumor thickness was large enough to place a region of interest of at least 0,05 cm^2^ into the tumor tissue on MDCT images for Hounsfield unit measurements
- Tumor volume and thickness were identical on arterial and late phase images according to mesothelioma modified RECIST criteria in case MDCTs were not done on the same day
- Diagnosis of MPM was confirmed by histology or unequivocal consensus statement of pneumologist, oncologist and radiologist in case histology was not available

**Fig. 1:**
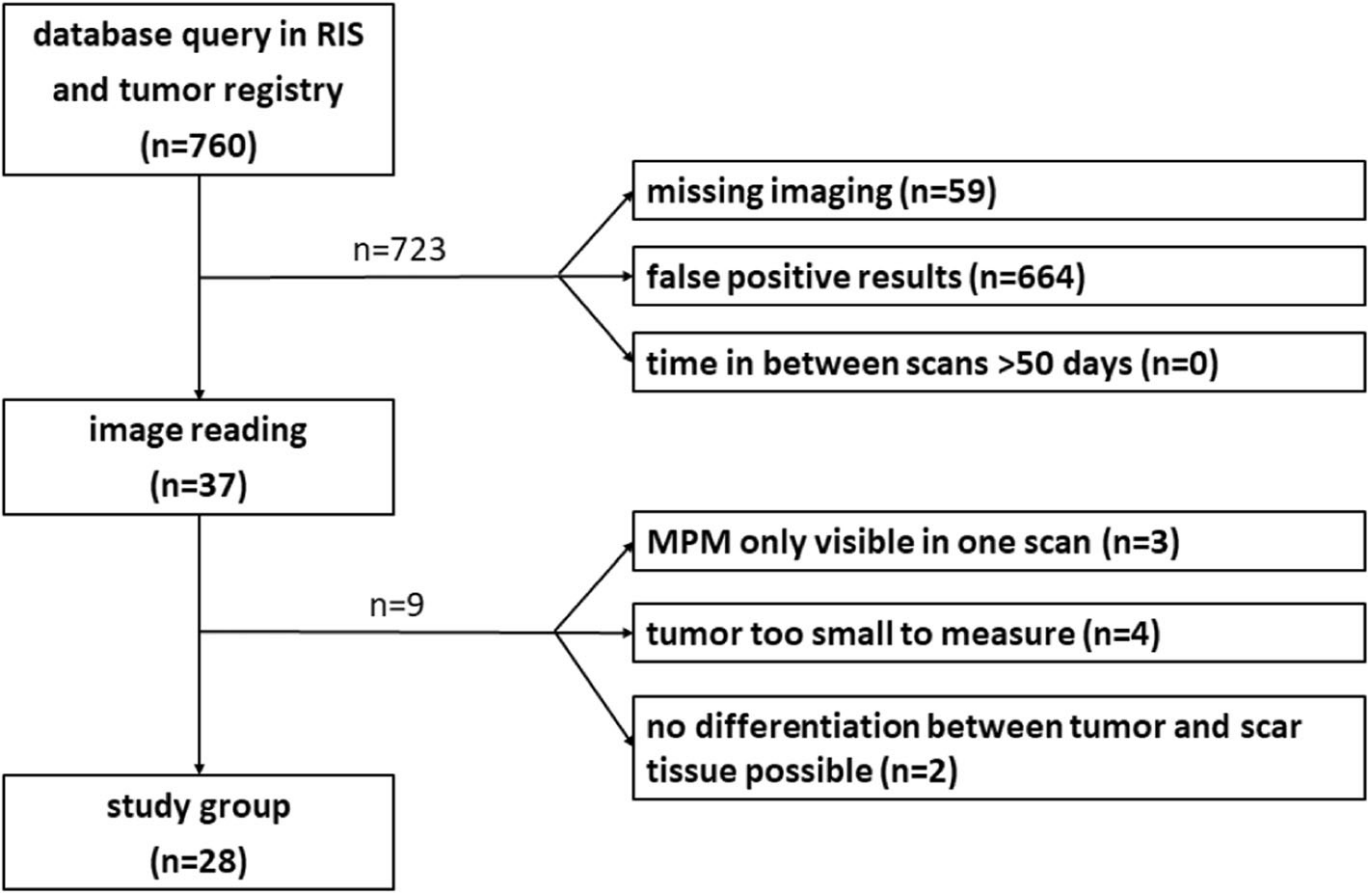
flow chart patient selection

The final study group consisted of 28 patients. A flow chart, summarizing the patient selection workflow is presented in Fig. 1.

### Data evaluation

Epidemiological parameters were acquired from the patients’ charts. Scan parameters were determined by reviewing the CT scans’ DICOM-Dataset and scan protocol. Scan delay after contrast injection was evaluated by checking the images for established characteristics of arterial and portal venous phase, for example contrast agent in the aorta but not in the venous vessels in arterial phase.

### Image analysis

#### Subjective analysis

Soft tissue reconstructions of corresponding scans in arterial and late phase were opened simultaneously on a PACS workstation. Window-settings were set to identical values. Subjective analysis was performed by two radiologists (senior thoracic radiologist with 16 years of experience and resident with 4 years of experience) independently to detect possible differences in tumor perception based on differences in reader experience. Arterial phase and late phase scans were compared regarding the conspicuity of MPM by rating the contrast of tumor tissue in comparison to the surrounding healthy tissue. Rating was done on a 3-point ordinal scale (− 1: tumor better visible in arterial phase, 0: tumor visibility equal among phases, + 1: tumor better visible in late phase). The complete data set of each scan was provided, but only tumor lesions located in the overlapping field of view of both scans were considered for analysis. Blinding of the readers regarding contrast phase was done by hiding vessels and viscera by a third observer who was not involved in image analysis.

#### Objective analysis

Density of the tumor in Hounsfield-Units (HU) was measured by placing a region of interest (ROI) inside the tumor. The largest tumor nodule was chosen. The location for the ROI placement was determined by the two observers in consensus as was the drawing in a separate session. The ROI had to measure at least 0.05 cm^2^ in size but was drawn as large as possible as long as the borders of the ROI were definitely within the tumor with a distance to the tumor margin of at least 2 mm. ROIs had to be identical in size and position for arterial phase and late phase scan. The same technique was applied to measure the density of immediately adjacent, non-tumorous soft tissue like mediastinal fat or thoracic wall, but not lung tissue or pleural effusion. Each measurement was checked for repeatability. HU values were regarded as valid only if the HU value of the original measurement and the repetition were not more than 5.0% apart.

The following values were calculated from the above outlined HU measurements:

- HU_diffLes_ = density of tumorous lesion in arterial phase (HU_earlyLes_) - density of tumorous lesion in late phase (HU_lateLes_)
- HU_diffAdj_ = density of adjacent soft tissue in arterial phase (HU_earlyAdj_) – density of adjacent soft tissue in late phase (HU_lateAdj_)
- HU_diffContrast_ = HU_diffLes_ – HU_diffAdj_

HU_diffContrast_ represents the difference in contrast between tumor and adjacent soft tissue between arterial phase and late phase.

### Statistics

For statistical analysis of HU-measurements, paired t-test for related samples was applied whenever measurements were normally distributed.

To exclude any confounder effects by epidemiologic factors or differing technical parameters between arterial and late phase images multiple regression analysis using general linear models was performed including the following parameters: histological subtype of MPM, gender, chemotherapy prior to the CT scans, tube voltage, tube current-time-product and slice thickness.

All data were analysed using SPSS (IBM SPSS Statistics for Macintosh, version 22). Results were regarded statistically significant if *p* was < 0.05 and highly statistically significant if *p* was < 0.01.

## Results

### Epidemiology

24 (85.7%) patients were male and 4 (14.3%) were female. Average age at time of diagnosis was 66.3 years (standard deviation (SD) 8.2 years), varying from 51 to 78 years. In 16 patients (57.1%), prior exposure to asbestos was confirmed. For 1 case (3.6%), asbestos-exposure could be ruled out. In 11 cases (39.3%), no reliable information in this regard was available. For 26 patients the arterial and late phase scans were done on the same day. For one patient arterial and late phase scans were done on two consecutive days and for one patient the scans were 35 days apart.

In 23 patients, data for the histological subtype of MPM was available showing epitheloid pleural mesothelioma in 16 patients (57.1%) and non-epitheloid pleural mesothelioma in 7 patients (25.0%).

### Subjective analysis

**Fig. 2:**
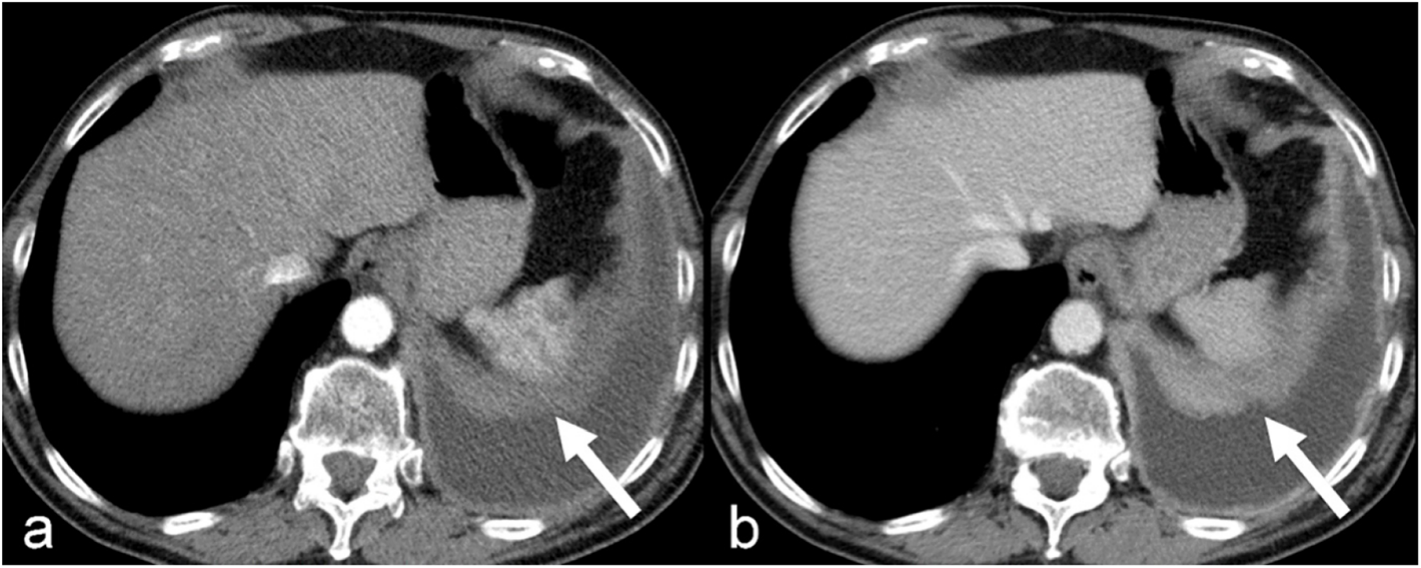
The tumor (arrows) shows considerably better conspicuity in late phase (**b**) compared to arterial phase (**a**). Window settings have been set to identical values, both images have identical technical parameters regarding tube voltage and slice-thickness

In all 28 cases and for both readers, MPM was better visible on late phase compared with arterial phase images. An example for a case, in which the tumor lesion showed considerably better conspicuity in late phase compared to early phase is presented in Fig. 2.

### Objective analysis

HU_earlyLes_ was in average 56.7 HU (SD 18.1 HU), HU_lateLes_ in average 75.4 HU (SD 22.9 HU). Hence, HU_diffLes_ was 18.6 HU (SD 10.1 HU) with the difference being statistically significant (*p* < 0.001).

Density of the surrounding soft tissue was − 17.4 HU (SD 47.5 HU) in arterial phase (HU_earlyAdj_) and − 17.3 HU (SD 12.1 HU) in late phase (HU_lateAdj_), resulting in an average difference (HU_diffAdj_) of 0.1 HU (SD 5.6 HU) between arterial phase and late phase with *p* = 0.92.

Average difference of contrast between tumor and adjacent soft tissue in arterial phase and late phase (HU_diffContrast_) was 18.5 HU (SD 10.6 HU) with *p* < 0.001. The results are summarized in Table 1.

**Table 1:** Results of the objective analysis

Parameter	Value (SD)	Significance (t-test)
HU_earlyLes_	56.7 HU (18.1 HU)	
HU_lateLes_	75.4 HU (22.9 HU)	
HU_earlyAdj_	−17.4 HU (47.5 HU)	
HU_lateAdj_	−17.3 HU (12.1 HU)	
**Parameter differences**		
HU_diffLes_	18.6 HU (10.1 HU)	*p* < 0.001
HU_diffAdj_	0.1 HU (5.6 HU)	*p* = 0.92
HU_diffContrast_	18.5 HU (10.6 HU)	*p* < 0.001

Technical and epidemiological data were as follows:

- Tube voltage

Tube voltage varied between 100 kV and 140 kV in arterial phase (mean 121.8 kV, SD 8.6 kV) and 100 kV and 130 kV in late phase (mean 117.8 kV, SD 7.3 kV). In 22 cases (79%), tube voltage was identical in arterial phase and late phase. In 6 cases (21%), tube voltage was higher in arterial phase than in late phase.

- Tube-current-time-product

For one patient, no conclusive data for the tube-current-time-product was available. Mean tube-current-time-product for arterial phase was 104.7 mAs (SD 54.6 mAs) with a range from 13 to 228 mAs. Mean tube-current-time-product for late phase was 148.8 mAs (SD 88.6 mAs), ranging from 13 to 385 mAs.

- Slice thickness

Slice thickness varied between 1.25 mm and 8.00 mm with an average of 5.30 mm (SD 1.80 mm) in arterial phase scans and 4.50 mm (SD 1.20 mm) in late phase scans. Slice thickness was 1.25 mm, 2.00 mm and 2.50 mm in one patient each in arterial as well as in late phase. In two cases, slice thickness was 3.75 mm in arterial and late phase. In 15 cases, slice thickness in arterial phase was 5.00 mm, in two cases 6.00 mm and in 6 cases 8.00 mm. In 22 cases, slice thickness in late phase was 5 mm, in one case 6 mm. In 20 cases (71.4%), slice thickness was identical between arterial phase and late phase. In 8 cases (28.6%), slice thickness was thinner in arterial phase.

- Tumor histology

In 23 patients (82.1%), the histological subtype of MPM was available. Histology showed an epitheloid type MPM in 16 patients (69.6%) and a biphasic, sarcomatoid or rare subtype of MPM in 7 patients (30.4%).

- Chemotherapy

For one patient, no information about the therapeutic regime was available. 14 Patients (51.9%) had been treated with chemotherapy prior to CT scans. 13 patients (48.1%) had not received any chemotherapy prior to imaging.

Multiple linear regression analysis using a general linear model with HU_diffContrast_ as dependant variable and contrast phase, tube voltage, tube-current-time-product and slice thickness as quantitative predictors was performed to test possible confounding because of technical parameters. Contrast phase (*p* < 0.001) and tube voltage (*p* < 0.001) proved to be highly significant independent predictors for tumor contrast. In a second, third and fourth general linear model confounding because of epidemiological parameters was tested with contrast phase as quantitative predictor and tumor entity as well as chemotherapy and gender as two-level factors. All three epidemiological parameters were no significant predictor for tumor contrast. Results of the multiple regression analysis are summarized in Table 2.

**Table 2:** Results of the multiple regression analysis using general linear models

	**adjusted mean (95% CI)**	**significance**
**contrast arterial phase (HU)**	76.6 (58.9, 94.2)	
**contrast late phase (HU)**	92.7 (75.1, 110.3)	
**difference in contrast (HU)**	16.1 (14.0, 18.2)	*p* < 0.001
	**correlation coefficient (95% CI)**	
**tube voltage (kV)**	−1.05 (−1.30, −0.80)	*p* < 0.001
**tube-current-time-product (mAs)**	−0.014 (− 0.045, 0.017)	*p* = 0.369
**slice thickness (mm)**	1.35 (− 0.06, 2.75)	*p* = 0.059
**epitheloid MPM (*n*** **= 23)**	−29.5 (− 64.2, 5.16)	*p* = 0.091
**chemotherapy prior to CT (*n*** **= 27)**	14.8 (− 18.2, 47.7)	*p* = 0.366
**female gender (*n*** **= 4)**	0.047 (− 10.1, 12.9)	*p* = 0.803

## Discussion

Early diagnosis of MPM is of vital importance for patient’s prognosis [3, 4]. Median survival rate for stage 1 MPM is 22.2 months compared to 14.9 months in patients with stage 4 MPM [17]. Contrast-MDCT of the chest is the imaging modality of choice for detection and staging of MPM. Even in contrast enhanced CT differentiation of MPM from surrounding soft tissue can be very difficult. Hence especially in early stages of MPM with tumor lesions being small, optimization of contrast between tumor and the surrounding soft tissue is vital to improve tumor conspicuity and consecutively detectability in CT-scans.

Empirical data and our own experience suggest, that a longer delay between intravenous administration of contrast agent and start of the scan results in better conspicuity of pleural pathologies like malignancies or inflammatory processes compared to the around 35 s delay routinely used in most protocols for chest MDCT [11]. However, most of these studies compared data from interindividual scans only [11–13]. One study only compared pleural enhancement on chest CT intraindividually on arterial phase and delayed phase scans in patients with malignant pleural effusion [16]. The authors, however, grouped several pleural malignancies together and focussed rather on subjective image analysis than on objective evaluation. Moreover, the study has several limitations: Despite concentrating on subjective image analysis only one selected image per scan was presented to the readers which limits power. Blinding of the readers regarding contrast phase was done by hiding vessels and viscera. However, experienced radiologists are still able to suggest contrast phase. Furthermore, subgroup analyses were not performed although technical parameters differed between phases. Nevertheless, the results of the study supported the hypothesis that conspicuity of pleural malignancy benefits from late phase imaging. A recent study analysed contrast enhancement dynamics of MPM in MRI [18]. The results of the study demonstrate that optimal contrast enhancement of MPM in MRI is likely to occur 2.5–5 min following intravenous contrast agent administration. These data again support that MPM enhances rather late. However, contrast and imaging dynamics of MRI and CT are not identical.

The present study is the first to analyse and compare conspicuity of MPM in MDCT between arterial phase and late phase intraindividually by subjective and objective evaluation. We found that by subjective assessment MPM was better visible in late phase for both the experienced and unexperienced radiologist. The subjective impression was confirmed by objective measurements of tumor density and contrast to surrounding tissue in arterial phase in comparison with late phase.

Due to the retrospective nature of this study differences regarding technical CT parameters between arterial and late phase images and some inhomogeneities of the study group were unavoidable. To control for confounding effects produced by these discrepancies, we conducted a multiple regression analysis using general linear models. Among all tested parameters only contrast phase and tube voltage were independent predictors of MPM density and contrast to surrounding tissue. Influence of tube voltage is supposedly caused by the fact that in 6/28 cases tube voltage was lower in late compared to arterial phase. Lower tube voltage increases image contrast at the expense of image noise. The study results support the hypothesis that apart from adapting the scan delay choosing rather low tube voltages and optionally controlling the increasing noise with iterative reconstruction techniques might further enhance tumor conspicuity of MPMs on CT images. Increasing the amount of contrast material delivered might further enhance MPM conspicuity. A study group demonstrated that pleural enhancement in 40 patients with suspected pleural disease was higher (83 HU) when delivering 45 g iodine compared to 30 g iodine (59 HU) at a scan delay of 60 s [12]. In the present study an intraindividual comparison was done with 26/28 examinations being two phase chest-abdominal scans with a single contrast injection. Thus, evaluation of different contrast protocols was not possible.

The study has limitations. First, the number of cases (*n* = 28) was rather small despite searching a time frame of 12 years. This is due to the low prevalence and incidence of MPM which per definition is an orphan disease. The strict selection criteria of our study design additionally limited the number of eligible patients. Still, the results were highly significant emphasizing the large effect of increasing scan delay and lowering tube voltage. Another limitation of our study is, that the exact scan delays were not documented in the scan protocol or radiology report and imaging protocols might have changed during the time span of 12 years applied for patient recruitment. However, correct phases were ensured by checking the images for established characteristics of arterial and portal venous phase. Evaluation of further scan delays apart from arterial and portal venous phase would be desirable especially considering the above-mentioned results of MR-imaging showing the best delay being > 2.5 min [18]. However, when studying the results of the MR trial in detail, there was not much of increasing intensity beyond 70 s scan delay. Still, the focus of future studies should be evaluating different delays, iodine delivery rates and iodine doses. Performing a prospective study comparing several subgroups interindividually would probably require a multicenter approach or long recruitment periods due to the low incidence of MPM. Intraindividual comparisons on the other hand would impose radiation dose issues. Another limitation of the present study is the fact that it could not be investigated whether an improved conspicuity of MPM by late phase imaging has influence on patient prognosis. Furthermore, no comparison with other imaging techniques such as MRI and PET-CT was made, which could be a possible topic for future studies.

## Conclusions

In conclusion, the results of this study suggest that late phase imaging may improve the diagnostic accuracy for detection of MPM on chest MDCT. Hence, modification of the routinely done arterial phase chest imaging CT-protocol should be discussed when MPM is suspected. Further study is warranted to evaluate if late phase imaging affects patient prognosis.

## Data Availability

The datasets used and/or analysed during the current study are available from the corresponding author on reasonable request.

## Abbreviations

kV: Kilovolt
mAs: Milliampere Seconds
MDCT: Multi-detector-computed-tomography
MPM: Malignant pleural mesothelioma
HU: Hounsfield units
ROI: Region of interest
SD: Standard deviation
